# HNRNPC, a predictor of prognosis and immunotherapy response based on bioinformatics analysis, is related to proliferation and invasion of NSCLC cells

**DOI:** 10.1186/s12931-022-02227-y

**Published:** 2022-12-19

**Authors:** Zhuoyu Gu, Yang Yang, Qian Ma, Hui Wang, Song Zhao, Yu Qi, Yixin Li

**Affiliations:** 1 Department of Thoracic Surgery, The First Affiliated Hospital of Zhengzhou University, Zhengzhou University, Zhengzhou, 450052 China; 2 Department of Clinical Oncology, The First Affiliated Hospital of Zhengzhou University, Zhengzhou University, Zhengzhou, 450052 China; 3 Post-Doctoral Station of Clinical Medicine, The First Affiliated Hospital of Zhengzhou University, Zhengzhou University, Zhengzhou, 450052 China

**Keywords:** NSCLC, m6A methylation, HNRNPC, Proliferation, Invasion

## Abstract

**Background:**

Little is known about the relationship between N6-methyladenosine (m6A)-related genes and tumor immune microenvironment (TIME) in non-small cell lung cancer (NSCLC). It is unclear which m6A regulators are essential for NSCLC progression. The aim of this work was to excavate the role of m6A-related genes in the TIME and progression of NSCLC.

**Methods:**

Based on bioinformatics analysis, heterogeneous nuclear ribonucleoprotein C (HNRNPC) was considered as the most influential m6A regulator. Further study was investigated using patient samples, stable cell lines, and xenograft mice models.

**Results:**

The differentially expressed profiles of m6A-related genes were established in NSCLC, and the NSCLC samples were clustered into two subtypes with different immune infiltration and survival time. Next, we found that the risk score (RS) based on m6A-related genes was a predictor of prognosis and immunotherapy response for NSCLC, in which HNRNPC was considered as the most influential m6A regulator. In NSCLC patients, we confirmed that HNRNPC predicted poor prognosis and correlated with tumor invasion and lymph node metastasis. RNA-seq data revealed that HNRNPC was involved in cell growth, cell migration, extracellular matrix organization and angiogenesis. In vitro, we verified that HNRNPC knockdown attenuated the cell proliferation, clonogenicity, invasion and migration. In vivo*,* HNRNPC knockdown inhibited the tumor growth and lung metastasis. Additionally, HNRNPC knockdown was associated with high CD8 + T cell infiltration, along with elevated CD4 + T cell infiltration, collagen production and angiogenesis.

**Conclusions:**

M6A regulator HNRNPC, a predictor of prognosis and immunotherapy response based on bioinformatics analysis, is related to proliferation and invasion of NSCLC cells.

**Supplementary Information:**

The online version contains supplementary material available at 10.1186/s12931-022-02227-y.

## Highlights

M6A-related genes with prognosis, PD-L1 and TIME in NSCLC were first assessed.

HNRNPC was the most influential m6A regulator predicted prognosis and immunotherapy response.

HNRNPC predicted poor prognosis and correlated with tumor invasion and lymph node metastasis.

HNRNPC promoted proliferation and invasion of NSCLC cells and was associated with CD8 + T cell infiltration in TIME.

**Supplementary Information:**

The online version contains supplementary material available at 10.1186/s12931-022-02227-y.

## Introduction

Lung cancer (LC) is one of the most aggressive malignancies in the world. As a leading cause of cancer-related mortality worldwide, LC can be divided into non-small cell lung cancer (NSCLC) and small cell lung cancer [1]. Nowadays, NSCLC constitutes the majority of LC patients, in which lung adenocarcinoma (LUAD) and lung squamous cell carcinoma (LUSC) are the most prevalent pathological subtypes. Despite the notable progress in NSCLC therapy, effective treatment remains to a challenge. Difficulty in early detection, high metastasis rates, resistance to radiotherapy and chemotherapy, and lack of systematic treatment are the main reasons for its high mortality rate [2]. Therefore, achieving detailed molecular mechanisms and identifying new molecular markers are extremely essential for diagnosis and treatment of NSCLC.

N6-methyladenosine (m6A) methylation is the most common mRNA modification in the N6 position of adenosine and plays a regulatory role in cellular and physiological process by altering gene expression. Emerging evidence has determined the critical role of m6A in cancer pathogenesis and progression [3, 4]. The function of m6A is mediated by writers (METTL3/14/15, WTAP, KIAA1429, RBM15 and ZC3H13), erasers (FTO and ALKBH5), and readers (YTHDC1/2, YTHDF1/2/3, IGF2BPs, HNRNPA2/B1 and HNRNPC) or their interactions. It is reported that ALKBH5 accelerates the progression of glioblastoma stem cells (GSCs) by increasing FOXM1 expression through m6A modification [5]. The overexpression of METTL3 contributes to the development of acute myeloid leukemia by increasing m6A modification of SP1 and activating MYB/MYC signaling pathway [6]. Hypoxia induces ALKBH5 expression in breast cancer cells, which reduces the m6A modification of NANOG and promotes the initiation and metastasis of breast cancer [7]. However, to date, it is unclear which m6A regulators are necessary and essential for NSCLC progression, and their diverse expression patterns and prognostic values are rarely reported.

Recent advances have been achieved in the immune therapy for NSCLC, such as the development of immune-checkpoint inhibitors, especially the programmed cell death–ligand 1 (PD-L1) inhibitors [8]. Importantly, the tumor immune microenvironment (TIME) characterized by immune cell infiltration and PD-L1 expression in tumor cells plays a key role in the initiation and prognosis of [9]. However, little is known about the relationship between m6A methylation-related genes and TIME in NSCLC. Therefore, in the present study, we investigated the expression profiles of m6A-related genes in NSCLC samples and controls. The correlations between m6A-related genes with prognosis, PD-L1 and TIME in NSCLC were assessed.

HNRNPC, named as heterogeneous nuclear ribonucleoproteins C1/C2, is a 306 amino acid protein and localizes in the nucleus [10]. HNRNPC plays a role in the early steps of spliceosome assembly and pre-mRNA splicing [11]. Furthermore, HNRNPC modulates the stability and the level of translation of bound molecules [12]. Recently, it is reported that m6A affects the secondary structure of RNA, whereas HNRNPC can regulate mRNA abundance and splicing after recognizing m6A, which is called the “m6A switch” [13]. However, to date, the role of HNRNPC in the prognosis and progression of NSCLC is unclear. In this study, we found that m6A-related genes could predict prognosis and immunotherapy response for NSCLC, in which HNRNPC was considered as the most influential m6A regulator. Furthermore, HNRNPC was upregulated and correlated with poor prognosis in NSCLC patients. In addition, we identified that HNRNPC was markedly associated with proliferation and invasion of NSCLC cells. The aim of this work was to excavate the role of m6A-related genes in the TIME and progression of NSCLC.

## Methods

### Datasets and preprocessing

The bioinformatics databases used in this study were shown in Additional file 1. The gene expression datasets of 585 LUAD samples and 550 LUSC samples were downloaded from The Cancer Genome Atlas (TCGA) database (https://portal.gdc.cancer.gov/) based on Illumina HiSeq 2000 RNA Sequencing. Meanwhile, the corresponding clinical information was downloaded. After checking the expression data and the clinical information, 994 NSCLC samples and 107 normal counterparts with complete clinical information were included in this study and set as the training dataset. Since the LUAD data and LUSC data were derived from different batches, SVA package version 3.38.0 in R3.6.1, which contains functions for removing batch effects and other unwanted variation in high-throughput experiment, was utilized to process the 994 NSCLC data (http://www.bioconductor.org/packages/release/bioc/html/sva.html). The profile of the analyzed 994 NSCLC samples and their information were shown in Additional file 2.

In addition, the GSE50081 dataset was downloaded from Gene Expression Omnibus database (https://www.ncbi.nlm.nih.gov/geo/). This dataset was deposited by Pintilie et al. [14], including 181 NSCLC samples with clinical information. This dataset was sequenced based on the platform of GPL570 Affymetrix Human Genome U133 Plus 2.0 Array and served as the validation dataset.

### Differential gene expression analysis

Based on references, 22 recognized m6A RNA methylation related genes were obtained, including 9 methyltransferases genes (METTL3, METTL14, METTL15, WTAP, VIRMA, RBM15, RBM15B, KIAA1429, and ZC3H13), 2 demethylases genes (FTO and ALKBH5) and 11 m6A binding genes (YTHDC1, YTHDC2, IGF2BP1, IGF2BP2, IGF2BP3, YTHDF1, YTHDF2, YTHDF3, HNRNPA2B1, and HNRNPC). The expression profiles of these 22 genes were collected from TCGA dataset and their expression in NSCLC samples and normal counterparts were compared by *t* test in R 3.6.1 with cutoff value of *P* < 0.05. The differentially expressed methylation genes (DEMGs) were subjected to hierarchical clustering analysis according to centered pearson correlation algorithm, with the application of Pheatmap Version 1.0.8 in R3.6.1 [15] (https://cran.r-project.org/web/packages/pheatmap/index.html).

### Consensus clustering of DEMGs and survival analysis of subtypes

To further understand the biological features of DEMGs, the TCGA NSCLC samples were distinctly classified into two subtypes, defined as subtype 1 and subtype 2. The procedure was based on the expression level of the DEMGs using ConsensusClusterPlus package version 1.54.0 (http://www.bioconductor.org/packages/release/bioc/html/ConsensusClusterPlus.html) in R 3.6.1, which is an open-source software for unsupervised class discovery [16]. The cluster procedure is described in detail in previous study [16]. Briefly, the RNA sequencing (RNA-seq) data of TCGA NSCLC samples was input to the package. The subtypes were obtained by setting 80% item resampling, a maximum evaluated *k* of 20 and 1000 repetitions.

Kaplan–Meier survival analysis was performed to evaluate the correlation of survival time and different subtypes by using survival package version 2.41-1 in R 3.6.1 (http://bioconductor.org/packages/survivalr/). The clinical information of different subtypes was compared, such as age, gender, pathologic stage, metastasis, treatment, and recurrence.

### Correlation between subgroups and TIME

TIME is specific for different tumors and is correlated with the initiation, development, and metastasis of cancers. Immune infiltrating cells are part of tumor microenvironment that play critical role in cancer progression and outcome [17]. The immune cell fractions of NSCLC samples were evaluated by CIBERSORT (https://cibersortx.stanford.edu/) as described in previous study [18, 19]. Briefly, the RNA-seq data of TCGA NSCLC samples and a “signature matrix” containing signature genes for cell subsets of 22 types of immune cells were used as input. “Permutations” was set as 100 and the options of “disable quantile normalization” was checked. After running, the proportions of the immune cells for each sample were obtained. The proportions of different immune cells were compared between subtypes by *t* test in R 3.6.1 with *P* < 0.05 as threshold.

The ESTIMATE (Estimation of Stromal and Immune cells in Malignant Tumor tissues using Expression data) is a tool for predicting tumor purity using gene expression data, generating three scores, including stromal score, immune score, and ESTIMATE score. As described previously, the immune scores of each tumor sample were calculated by ESTIMATE package (https://sourceforge.net/projects/estimateproject/) [20]. The difference between immune scores was analyzed by *t* test in R 3.6.1 with *P* < 0.05 as the threshold. The subgroups were divided into high TIME group and low TIME group according to the distribution of immune scores of all samples.

Based on the gene expression profiles of all the included tumor samples, the Kyoto Encyclopedia of Genes and Genomes (KEGG) pathways significantly related with TIME were analyzed by GSEA (Gene Set Enrichment Analysis, http://software.broadinstitute.org/gsea/index.jsp) [21]. The adjusted *P* value (FDR, false discovery rate) < 0.05 was set as the cutoff value.

### Analysis of PD-L1 expression

The expression levels of PD-L1 between NSCLC samples and normal counterparts and between different subtypes were analyzed by *t* test function in R 3.6.1. The correlation between PD-L1 and DEMGs were analyzed by cor function in R 3.6.1.

### Prognostic risk factor model construction

To screen the DEMGs with potential to predict prognosis, the DEMGs were subjected to least absolute shrinkage and selection operator (LASSO) regression analysis by using lars package Version 1.2 (https://cran.r-project.org/web/packages/lars/index.html) [22] in R 3.6.1. The optimal lambda value was determined by 10 cross-validations. Then, the optimal DEMGs related with prognosis was selected using multivariate Cox proportional hazard regression analysis and a prognostic signature of risk score (RS) was established as follows: RS = ∑Coef_genes_ × Exp_genes._ Coef_genes_ indicates LASSO coefficient of target genes, Exp_genes_ represents expression level of a given gene in TCGA dataset.

### Evaluation of RS in predicting prognosis

RS values of the optimal DEMGs in TCGA training dataset and GESE50081 validation dataset were calculated, respectively. Based on the median of RS value, samples were divided into high RS group (RS > median value) and low RS group (RS < median score). We performed Kaplan–Meier survival analysis to evaluate the survival of patients in high and low RS groups and difference of survival time between groups was compared by log-rank test. Receiver operating characteristic (ROC) curves for 1-year, 3-year and 5-year survival was drawn. Then, the independent prognostic value of RS in TCGA dataset was detected by univariate and multivariate Cox regression analysis with the application of survival package Version 2.41-1 [23]. Finally, the distributions of RS values in different subtypes and independent clinical factors were investigated.

### Correlation between RS value and TIME

The effect of m6A related genes in immune cell infiltration was measured by online tool of TIMER (Tumor Immune Estimation Resource) (https://cistrome.shinyapps.io/timer/) [24], which contained 6 types of immune infiltrated cells, such as B cells, CD4 + T cells, CD8 + T cell, neutrophils, macrophage and dendritic cells). Then, the relationship between different types of infiltrated immune cells and RS value was calculated based on TCGA samples.

### Patient samples

Fresh human tumor tissues and adjacent normal tissues were obtained from 8 patients diagnosed with NSCLC between May 2020 and April 2021 at the First Affiliated Hospital of Zhengzhou University. Written informed consent was obtained from each patient involved in the study, and the study was approved by the ethics committee of the First Affiliated Hospital of Zhengzhou University (2022-KY-0109-004). In addition, human NSCLC tissue microarray of 30 cases was purchased from Superchip (Shanghai, China). Medical records of all patients included information about age, gender, pathological grade, and TNM stage, and the patients were followed up for 8 years.

### Cell culture and construction of stable cell lines

The human NSCLC cell lines (A549, H1299, and PC-9) and mouse lung cancer cell line (LLC) were obtained and authenticated from the Cell Bank of Chinese Academy of Sciences (Shanghai, China). All cells were grown in RPMI-1640 media that had been supplemented with 10% fetal bovine serum (FBS), and the mixture was placed in an incubator with a humidified environment that contained 5% carbon dioxide. The lentiviruses of human HNRNPC knockdown (shHN1: 5′-3′ GCCTTCGTTCAGTATGTTAAT, shHN2: 5′-3′ CTGGATGATGATGATAATGAA), and mouse HNRNPC knockdown (shHN1: 5′-3′ GCCTTTGTCCAGTATGTTAAT) were purchased from Shanghai GenePharma Co. (Shanghai, China). Through the use of selection containing 1 μg/mL puromycin for 4 weeks, stable cell lines were generated.

### Western blotting (WB) and Flow cytometry (FC)

Proteins were separated via SDS-PAGE and transferred onto PVDF membranes. The membranes were blocked with 5% non-fat milk for 2 h at room temperature and subsequently incubated with primary and secondary antibodies, and the protein bands were detected via enhanced chemiluminescence. The primary antibodies used were as follows: HNRNPC (Abclonal, #A19137, 1:1000), GAPDH (Abclonal, #A19056, 1:1000), PCNA (Abclonal, #A0264, 1:1000), N-cadherin (Abclonal, #A19083, 1:1000). For FC analysis, single-cell suspensions prepared from tumor tissues were stained with the mouse-specific CD4 and CD8 antibodies (BD Biosciences). Finally, cells were collected on a FACSCalibur (BD Biosciences) and analyzed by FlowJo software.

### MTS and colony formation assays

To complete the MTS assay, the cells were plated at a density of 5000 cells/well. Following a seeding duration of 1–3 days, the cells were subjected to 40 min of incubation with MTS (Promega, Madison, WI) for 40 min at each of those time points. Additionally, with the aid of a microplate reader, the optical density (OD) was measured. To facilitate the formation of colonies, the cells were planted in 6-well plates at 500 cells/well. After incubating the cells for two weeks, 4% paraformaldehyde was utilized to fix them before staining them with crystal violet. Images of the colonies were captured and processed with Image-Pro Plus 6.0 (Media Cybernetics, MD, USA) to facilitate the counting of the total colonies.

### Scratch wound healing and transwell assays

The cells were grown in 6-well plates until they reached confluence, and afterward, a 10-μL pipette tip was used to scrape through the cell layer that was present in each well. After taking measurements of the gap widths at 0 h (width 1(w1)) and 24 h (width 2(w2)), the relative rate of migration was computed as follows: (w1 − w2)/w1 × 100%. To execute a transwell migration assay, Boyden chambers that included 24-well transwell plates were used (BD, USA). The top chambers that had been covered with Matrigel were then introduced with homogenous single-cell suspensions. Once 24 h elapsed, crystal violet was used to stain the cells that had invaded through the membrane in the bottom chambers. The number of cells in each of the five random fields was counted.

### RNA sequencing (RNA-seq)

Briefly, total RNA was extracted for quality control and obtaining RNA-seq loading samples. RNA-seq was performed as DGE on an Illumina HiSeq platform, and 50-bp paired-end reads were generated by BGI Co. (Shenzhen, China). The deep sequencing data were submitted to the NCBI Gene Expression Omnibus (GEO) under the accession number GSE199483 (H1299 cells transfected with control or HNRNPC shRNA).

### In vivo tumor model

To establish xenograft models, approximately 1 × 10^6^ LLC cells in 100 μL of PBS were subcutaneously injected into C57BL/6 mice. Tumor volumes were recorded every 4 days and calculated as follows: volume = (width^2^ × length)/2. After 25 days, the tumor xenografts were harvested, weighed and processed for IF staining. To establish lung metastasis models, 1.5 × 10^6^ H1299 cells in 100 μL of PBS were injected into female BALB/c nude mice via the tail vein. Both bioluminescence imaging and histological analysis were used to investigate lung metastasis. Using IVIS Spectrum (Perkin Elmer, USA) for quantitative luminescence analyses, the normalized fluorescence intensities of lung tissues were measured and directly represented as the average radiance (photon/ second/ cm^2^/ steradian, or p/s/cm^2^/sr). The areas of metastatic foci in histopathological images were digitalized and calculated by using ImageJ software (NIH, MD, USA) for metastatic intensity analyses and quantified as the lung metastasis area (mm^2^). Every experiment involving animals was undertaken in compliance with the institutional ethical standards for animal studies that had been adopted by the First Affiliated Hospital of Zhengzhou University.

### Immunohistochemical (IHC) and immunofluorescence (IF)

For IHC, tissue sections were incubated with primary antibodies overnight. The tissue sections were washed and incubated with specific secondary antibodies and stained with diaminobenzidine (DAB).

Next, the tissue slides were scored independently by two investigators. For scoring, A score between 0 and 300 was obtained by multiplying the proportion of tumor cells that were stained (ranging from 0 to 100%) by the intensity (ranging from 1 to 3). Kaplan–Meier survival curves were used to assess the prognostic value. For IF, cells were seeded onto coverslips in a 6-well plate. Subsequently, the cells were fixed with 4% paraformaldehyde, permeabilised with 0.5% Triton-X and blocked with bovine serum albumin (BSA). The cells were then incubated with primary antibodies overnight. The following day, the cells were rinsed, then treated with secondary antibodies that had been fluorescently tagged, and finally stained with 4,6-diamidino-2-phenylindole (DAPI). Finally, images were captured using a confocal microscope (Olympus, Japan) and analyzed by using ImageJ software. The primary antibodies used were as follows: HNRNPC (Abclonal, #A19137, 1:100), Collagen I (Abclonal, #A16891, 1:100), CD31 (BD, #550274, 1:200), CD4 (Abcam, #ab183685, 1:100) and CD8 (Abcam, #ab217344, 1:200).

### GEPIA, UALCAN and human protein atlas (HPA) datasets

The GEPIA (http://gepia.cancer-pku.cn) and UALCAN (http://ualcan.path.uab.edu) datasets of TCGA gene expression were used to analyze the expression of HNRNPC in LUAD or LUSC and the correlation between HNRNPC and PCNA/ N-cadherin in tumor tissues was calculated. The methods used for analysis were based on the online instructions provided by the website. The survival information of 994 clinical patients with LUAD and LUSC, including 398 females and 596 males, was obtained from the HPA database (https://www.proteinatlas.org/). The overall survival (OS) of patients with LUAD was assessed according to the online instructions.

### Statistical analysis

SPSS (version 17.0), a statistical software tool, was utilized to execute the necessary analyses of statistical data. The presentation of all of the data was in the form of mean ± SD. Either a two-tailed Student t-test or a variance analysis was employed to determine whether or not the differences were significant. The Spearman rank correlation analysis was performed to evaluate the correlation, and the Kaplan–Meier analysis was utilized to evaluate the OS curves. When *P* was < 0.05, statistical significance was attained.

## Results

### The differentially expressed profiles of 15 m6A-related genes were established in NSCLC, and NSCLC samples were clustered into two subtypes with significantly different immune infiltration and survival time

**Fig. 1:**
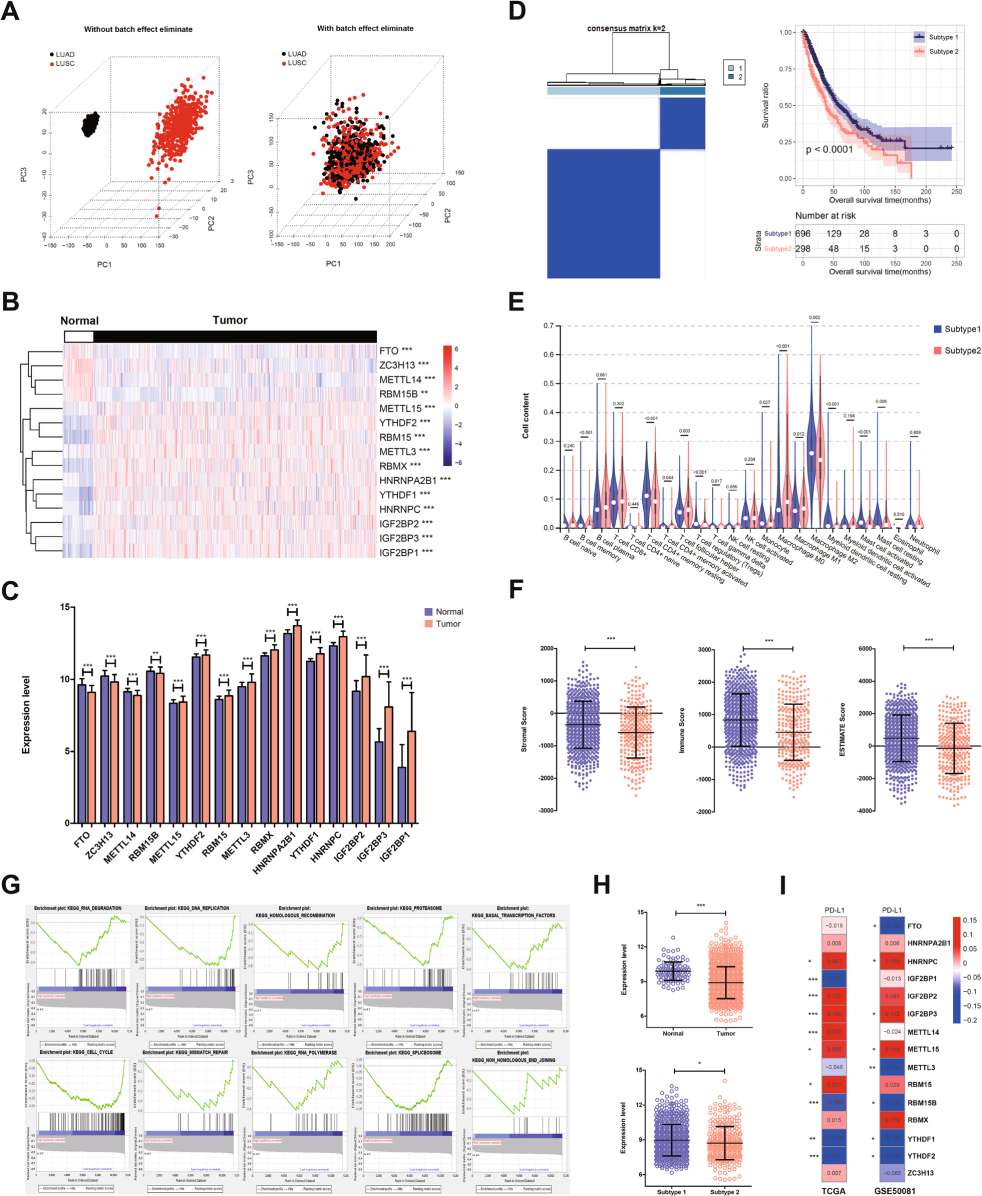
The differentially expressed profiles of 15 m6A-related genes were established in NSCLC, and NSCLC samples were clustered into two subtypes with significantly different immune infiltration and survival time. **a** The LUAD and LUSC samples were in different clusters before batch effect removal. After SVA-based batch effects elimination, LUAD and LUSC samples were mixed together, indicating the batch effects have been removed. **b** 15 m6A-related genes were significantly differentially expressed in NSCLC samples compared with that in normal controls. These 15 genes were regarded as DEMGs and the heatmap of the expression levels of DEMGs in the samples was shown. **c** Compared with normal counterparts, 4 DEMGs (FTO, ZC3H13, METTL14 and RBM15B) were significantly down-regulated in tumor samples, while the remaining 11 genes were upregulated in tumor samples. **d** Based on the expression level of the DEMGs, NSCLC samples were distinctly classified into two subtypes, defined as subtype 1 and subtype 2 (left). There were 696 samples in subtype 1 and 298 samples in subtype 2. Subtype 1 showed preferentially longer overall survival time than subtype 2 (right). **e** The infiltration levels of 13 immune cells were significantly different between subtypes. Subtype 1 displayed higher levels of B memory cells, T regulatory cell, gamma delta T cell, resting NK cell, Monocyte, M2 macrophage, activated mast cell, resting mast cell. **f** Subtype 1 was present with higher stromal score, immune score, and ESTIMATE score. Thus, subtype 1 with higher immune scores was defined as high TIME group, and subtype 2 was defined as the low TIME group. **g** Total 10 KEGG pathways were predicted to be closely related with high and low TIME. **h** The expression level of PD-L1 was significantly decreased in NSCLC samples. PD-L1 expression was higher in subtype 1 than that in subtype 2. **i** PD-L1 consistently exerted negative correlation with YTHDF1, YTHDF2, RBM15B, while showed positively correlation with HNRNPC, IGF2BP3, and METTL15. *P < 0.05, **P < 0.01, ***P < 0.001

The gene expression datasets of LUAD and LUSC samples were downloaded from TCGA database. The batch effects of LUAD and LUSC samples were eliminated by SVA package. The sample distribution before and after batch effect removal was visualized by principal component analysis. As shown in Fig. 1A, the LUAD and LUSC samples were in different clusters before batch effect removal. After SVA-based batch effects elimination, LUAD and LUSC samples were mixed together, indicating the batch effects have been removed (Fig. 1A).

The expression profiles of 22 m6A methylation-related genes in each sample were extracted from the TCGA dataset. The expression value of 20 m6A-related genes were found and 15 of them were significantly differentially expressed in NSCLC samples compared with that in normal controls (Additional file 3: Table S1). These 15 genes (YTHDF1, HNRNPC, IGF2BP3, HNRNPA2B1, RBMX, IGF2BP1, IGF2BP2, FTO, METTL14, ZC3H13, RBM15, METTL3, YTHDF2, METTL15 and RBM15B) were regarded as DEMGs. The heatmap of the expression levels of DEMGs in the samples was shown in Fig. 1B, and the distribution of expression levels in the tumor and control samples was shown in Fig. 1C. The 15 DEMGs could distinguish the tumor samples from the normal samples, indicating the reliability of the results (Fig. 1B). Compared with normal counterparts, 4 DEMGs (FTO, ZC3H13, METTL14 and RBM15B) were significantly down-regulated in tumor samples, while the remaining 11 genes were upregulated in tumor samples (Fig. 1C).

Based on the expression level of the DEMGs, NSCLC samples were distinctly classified into two subtypes, defined as subtype 1 and subtype 2, by consensus clustering with *k* = 2 (Fig. 1D). There were 696 samples in subtype 1 and 298 samples in subtype 2 (Additional file 4: Table S2). Subtype 1 showed preferentially longer overall survival time than subtype 2 (*P* < 0.0001, Fig. 1D). Then, the clinical characteristics between subtype 1 and subtype 2 were compared by chi-square test. There was significant difference on gender and pathologic stage (*P* < 0.05), while no significant difference regarding age, M/N/T stage, radiotherapy, and recurrence was found (*P* > 0.05, Table 1).

**Table 1:** The clinical characteristics were compared between subtype 1 and subtype 2

characteristics total cases	N of case 994	Subtype		P value
		Subtype 1 (N = 696)	Subtype 2 (N = 298)	
Age (years)				
≤ 60	264	181	83	5.826E-01
> 60	715	504	211	
Geneder				
Male	596	391	205	**2.323E-04**
Female	398	305	93	
Pathologic M				
M0	738	512	226	6.918E-01
M1	31	23	8	
Pathologic N				
N0	640	458	182	3.518E-01
N1	221	149	72	
N2	109	70	39	
N3	7	5	2	
Pathologic T				
T1	281	209	72	1.157E-01
T2	554	383	171	
T3	115	77	38	
T4	41	24	17	
Pathologic stage				
Stage I	510	375	135	**1.508E-02**
Stage II	277	188	89	
Stage III	163	99	64	
Stage IV	32	24	8	
Ratiotherapy				
Yes	110	79	31	9.980E-01
No	764	544	220	
Recurrence				
Yes	251	180	71	9.331E-01
No	561	399	162	

To evaluate the correlation between subtypes with TIME of NSCLC, immune cell fractions and immune scores were analyzed. The proportions of total 22 types of immune cells were predicted by CIBERSORT algorithm (Additional file 5: Table S3). The difference of immune cell fractions between subtype 1 and subtype 2 were analyzed by *t* test. As shown in Fig. 1E, the infiltration levels of 13 immune cells were significantly different between subtypes. Subtype 1 displayed higher levels of B memory cells, T regulatory cell, gamma delta T cell, resting NK cell, Monocyte, M2 macrophage, activated mast cell, resting mast cell, whereas subtype 2 was correlated with more CD4 + memory resting T cell, follicular helper T cell, activated Myeloid dendritic cell. The immune scores of each tumor sample were calculated by ESTIMATE package (Additional file 6: Table S4). As illustrated in Fig. 1F, subtype 1 was present with higher stromal score, immune score, and ESTIMATE score. Thus, subtype 1 with higher immune scores was defined as high TIME group, and subtype 2 was defined as the low TIME group. Total 10 KEGG pathways were predicted to be closely related with high and low TIME, such as RNA degradation, Homologous recombination, and DNA replication (Fig. 1G, Additional file 7: Table S5).

The expression levels of PD-L1 in all samples were obtained and differences between NSCLC samples and normal controls and between subtype 1 and subtype 2 were compared by *t* test. The expression level of PD-L1 was significantly decreased in NSCLC samples, compared with that in normal controls (*P* < 0.001). PD-L1 expression was higher in subtype 1 than that in subtype 2 (*P* < 0.05, Fig. 1H). Correlation analysis between PD-L1 expression and DEMGs expression in TCGA training dataset and GSE50081 validation dataset revealed that PD-L1 consistently exerted negative correlation with YTHDF1, YTHDF2, RBM15B, while showed positively correlation with HNRNPC, IGF2BP3, and METTL15 (Fig. 1I, Additional file 8: Table S6). Taken together, the differentially expressed profiles of 15 m6A-related genes were established in NSCLC, and NSCLC samples were clustered into two subtypes with significantly different immune infiltration and survival time.

### The risk score (RS) based on 5 m6A-related genes with excellent prognostic value was a predictor of prognosis and immunotherapy response for NSCLC, in which HNRNPC was considered as the most influential m6A regulator

**Fig. 2:**
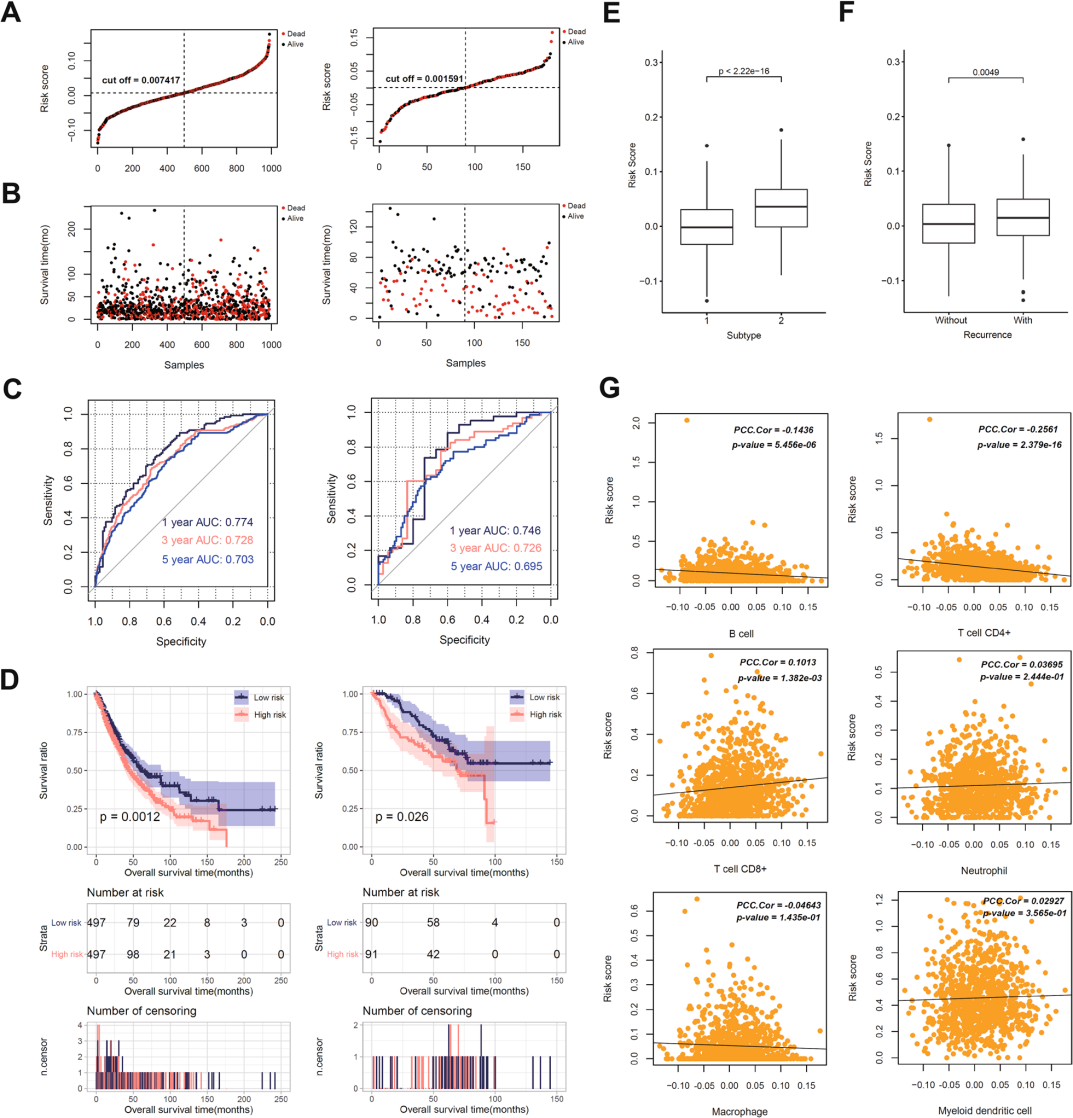
The RS based on 5 m6A-related genes with excellent prognostic value was a predictor of prognosis and immunotherapy response for NSCLC, in which HNRNPC was considered as the most influential m6A regulator. **a** The distribution of risk score (RS) in samples of The Cancer Genome Atlas (TCGA) dataset and GSE50081 validation dataset. **b** The distribution of overall survival status in samples of TCGA dataset and GSE50081 validation dataset. **c** ROC curve analysis of RS was performed. In TCGA dataset, the RS showed a good performance for prognostic prediction with area under ROC curves (AUCs) of 0.774, 0.7826, 0.703 for 1-, 3-, 5-year survival time. The AUCs for 1-, 3-, and 5-year survival time was 0.746, 0.726 and 0.695 in validation dataset. **d** Low RS group was correlated with longer survival time in both TCGA and GSE50081 datasets. **e** RS value was significantly different between the two subtypes. **f** RS value was significantly different between the patients with recurrence and without recurrence. **g** RS was positively correlated with infiltration of CD8 + T cell, while was negatively correlated with the infiltration levels of B cell and CD4 + T cell

Five DEMGs (HNRNPA2B1, HNRNPC, IGF2BP1, METTL3 and RBM15B) were selected as the optimal DEMGs for predicting the prognosis of NSCLC by using LASSO regression model. The LASSO regression coefficient of the five DEMGs is shown in Additional file 9: Table S7. Then, RS was calculated based on the LASSO regression coefficient and the expression level of optimal DEMGs in TCGA training dataset and GSE50081 validation dataset, separately (Additional file 10: Table S8). The formula was listed as follow: RS = (0.003612586) * ExpHNRNPA2B1 + (0.052669305) * ExpHNRNPC + (0.006522287) * ExpIGF2BP1 + (− 0.079231555) * ExpMETTL3 + (0.001102796) * ExpRBM15B. The distribution of RS, survival time and survival status in TCGA training and GSE50081 validation dataset was depicted in Fig. 2A and B. To evaluate the accuracy of predictive accuracy of the RS, we performed 1-, 3-, 5-year ROC curve analysis. In TCGA dataset, the RS showed a good performance for prognostic prediction with area under ROC curves (AUCs) of 0.774, 0.7826, 0.703 for 1-, 3-, 5-year survival time. The AUCs for 1-, 3-, and 5-year survival time was 0.746, 0.726 and 0.695 in validation dataset (Fig. 2C). Then, the samples in TCGA and GSE50081 dataset were divided into high and low risk group based on the RS median value. The correlation of RS with prognosis was assessed by Kaplan–Meier survival analysis. As shown in Fig. 2D, low RS group was correlated with longer survival time in both TCGA (*P* = 0.0012) and GSE50081 datasets (*P* = 0.026). Furthermore, univariable and multivariable cox regression analyses were preformed to determine whether RS was an independent prognostic factor for NSCLC by integrating with clinical characteristics. Multivariable analysis showed that the recurrence (*P* = 3.910E-07) and RS (*P* = 1.683E-02) were closely associated with prognosis of NSCLC patients (Table 2). In addition, RS was significantly different between the two subtypes (*P* < 2.22E-16, Fig. 2E), so was between with or without recurrence status (*P* = 0.0049, Fig. 2F).

**Table 2:** Uni-variable and multi-variable cox regression analysis were preformed to determine the independent prognostic factors for NSCLC

Clinical characteristics	Uni-variable cox		Multi-variable cox	
	HR (95% CI)	P value	HR (95% CI)	P value
Age (years, mean ± SD)	1.012 [1.001–1.023]	3.387E-02	1.013 [0.998–1.030]	9.860E-02
Gender (Male/Female)	1.414 [0.929–1.402]	2.074E-01	–	–
Pathologic M (M0/M1)	2.258 [1.432–3.561]	3.167E-04	0.879 [0.322–2.398]	8.005E-01
Pathologic N (N0/N1/N2/N3)	1.409 [1.240–1.602]	1.177E-07	0.991 [0.716–1.370]	9.542E-01
Pathologic T (T1/T2/T3/T4)	1.423 [1.254–1.615]	4.764E-08	1.176 [0.927–1.491]	1.828E-01
Pathologic stage (I/II/III/IV)	1.477 [1.329–1.643]	2.782E-13	1.291 [0.898–1.857]	1.676E-01
Radiation therapy (Yes/No/–)	1.593 [1.203–2.109]	1.043E-03	1.359 [0.936–1.973]	1.072E-01
Recurrence (Yes/No/–)	2.254 [1.789–2.840]	1.339E-12	2.053 [1.555–2.711]	**3.910E-07**
RS status (High/Low)	1.393 [1.139–1.705]	1.166E-03	1.224 [0.918–1.630]	**1.683E-02**

The fractions of six types of immune cells (B cell, CD4 + T cell, CD8 + T cell, neutrophil, macrophage, and myeloid dendritic cell) were predicted by TIMER online tool (Additional file 11: Table S9). The correlation between RS and infiltration of immune cells was analyzed by Pearson correlation analysis. Pearson correlation analysis showed that RS was positively correlated with infiltration of CD8 + T cell, while was negatively correlated with the infiltration levels of B cell and CD4 + T cell (Fig. 2G). As discussed above, HNRNPC and RBM15B were correlated with PD-L1 both in training dataset and validation dataset. Due to the higher coefficient in RS formula, HNRNPC was considered as the most influential m6A regulator. Altogether, the RS based on 5 m6A-related genes with excellent prognostic value was a predictor of prognosis and immunotherapy response for NSCLC, in which HNRNPC was focused and further studied.

### HNRNPC predicted poor prognosis in NSCLC patients and correlated with tumor invasion and lymph node metastasis

**Fig. 3:**
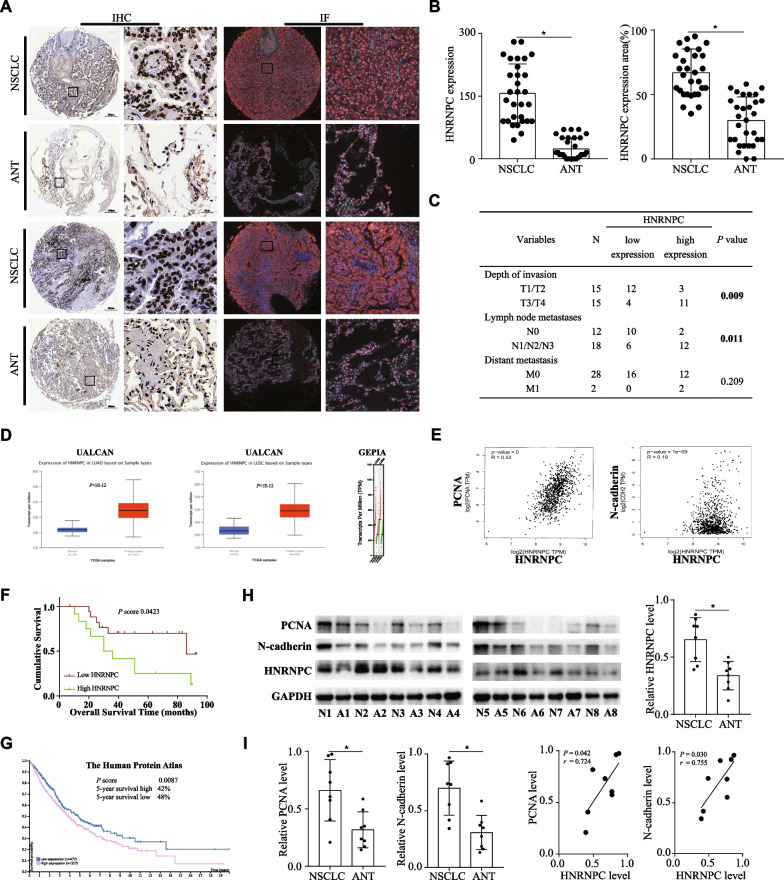
HNRNPC predicted poor prognosis in NSCLC patients and correlated with tumor invasion and lymph node metastasis. **a, b** The expression of HNRNPC was significantly increased in NSCLC tissues and HNRNPC was mainly localized to the nucleus (*n* = 30). **c** The relationship between HNRNPC and clinicopathological characteristics of NSCLC patients was analyzed. A correlation existed between higher HNRNPC expression and deeper tumor invasion, and a correlation between greater HNRNPC expression and an elevated probability of lymph node metastasis. **d** In UALCAN and GEPIA databases, the expression of HNRNPC was elevated in LUAD and LUSC tissues. **e** In LUAD and LUSC tissues of GEPIA database, further correlation analysis indicated a positive expression correlation between HNRNPC and PCNA, along with a positive expression correlation between HNRNPC and N-cadherin. **f** The survival analysis revealed that high HNRNPC expression in 30 tumor tissues predicted a poor prognosis for NSCLC patients. **g** In HPA database, the data also suggested that the NSCLC patients with high expression of HNRNPC had a shorter overall survival. **h** We collected 8 cases of NSCLC tissues and ANTs, and increased expression of HNRNPC was confirmed in NSCLC tissues (*n* = 8). **i** PCNA and N-cadherin were abundant in NSCLC tissues than ANTs. In NSCLC tissues, further correlation analysis also indicated a positive expression correlation between HNRNPC and PCNA, along with a positive expression correlation between HNRNPC and N-cadherin. *P < 0.05

Through immunohistochemistry (IHC) and immunofluorescence (IF) analysis, the expression of HNRNPC was firstly examined in tissue microarray of 30 paraffin-embedded NSCLC tissues and adjacent normal tissues (ANTs). Significantly, the expression of HNRNPC was increased in NSCLC tissues and HNRNPC was mainly localized to the nucleus (Fig. 3A, B). Furthermore, the relationship between HNRNPC and clinicopathological characteristics of NSCLC patients was analyzed. We discovered that a correlation existed between higher HNRNPC expression and deeper tumor invasion (*P* = 0.009), and a correlation between greater HNRNPC expression and an elevated probability of lymph node metastasis (*P* = 0.011) (Fig. 3C). In UALCAN and GEPIA databases, the data showed that the expression of HNRNPC was elevated in lung adenocarcinoma (LUAD) and lung squamous cell carcinoma (LUSC) tissues (Fig. 3D). PCNA and N-cadherin are the markers of cell proliferation and invasion. In LUAD and LUSC tissues of GEPIA database, further correlation analysis indicated a positive expression correlation between HNRNPC and PCNA (*r* = 0.53, *P* < 0.05), along with a positive expression correlation between HNRNPC and N-cadherin (*r* = 0.19, *P* < 0.05) (Fig. 3E).

Next, through the survival analysis, we revealed that high HNRNPC expression in 30 tumor tissues predicted a poor prognosis for NSCLC patients (*P* < 0.05) (Fig. 3F). In Human Protein Atlas (HPA) database, the data also suggested that the NSCLC patients with high expression of HNRNPC had a shorter overall survival (*P* < 0.05) (Fig. 3G). Meanwhile, we collected 8 cases of NSCLC tissues and ANTs, and increased expression of HNRNPC was confirmed in NSCLC tissues (Fig. 3H). Moreover, we found that PCNA and N-cadherin were abundant in NSCLC tissues than ANTs. In NSCLC tissues, further correlation analysis also indicated a positive expression correlation between HNRNPC and PCNA (*r* = 0.724, *P* < 0.05), along with a positive expression correlation between HNRNPC and N-cadherin (*r* = 0.755, *P* < 0.05) (Fig. 3I). Collectively, these data indicated that HNRNPC predicted poor prognosis in NSCLC patients and correlated with tumor invasion and lymph node metastasis.

### HNRNPC was involved in cell growth, cell migration, extracellular matrix organization and angiogenesis

**Fig. 4:**
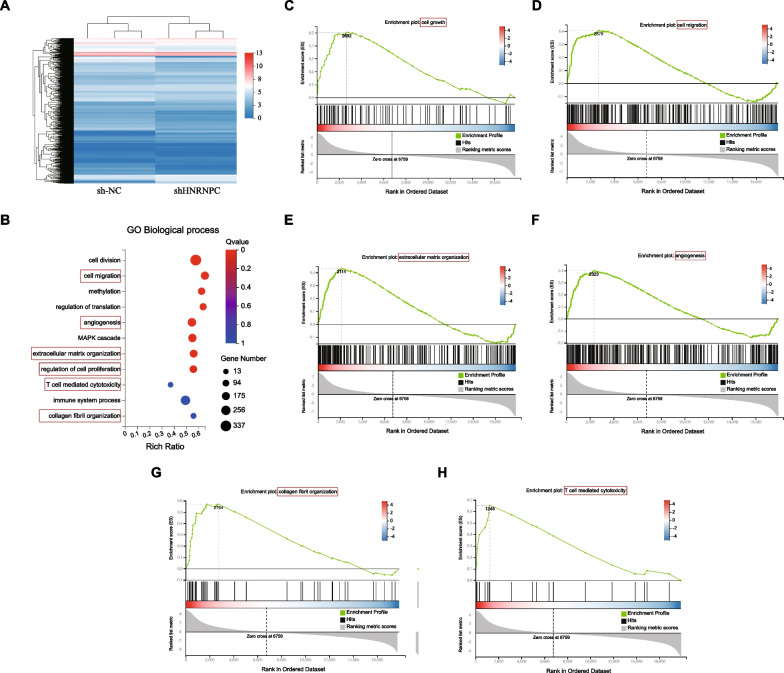
HNRNPC was involved in cell growth, cell migration, extracellular matrix organization and angiogenesis. **a** We constructed H1299 cells with stably knockdown of HNRNPC (shHNRNPC) and control cells (sh-NC). By RNA-seq analysis, 10,907 differentially expressed genes were identified, including 5653 upregulated and 5254 downregulated genes. **b** The GO analysis revealed that the differentially expressed genes were enriched in cell migration, angiogenesis, extracellular matrix organization, cell proliferation, T cell mediated cytotoxicity and collagen fibril organization. **c–h** The GSEA analysis demonstrated that the cell growth, cell migration, extracellular matrix organization, angiogenesis, collagen fibril organization and T cell mediated cytotoxicity were correlated with HNRNPC knockdown

Next, the function of HNRNPC in NSCLC was further explored. We constructed H1299 cells with stably knockdown of HNRNPC (shHNRNPC) and control cells (sh-NC). By RNA-seq analysis, 10,907 differentially expressed genes were identified, including 5653 upregulated and 5254 downregulated genes (Fig. 4A). The Gene Ontology (GO) analysis revealed that the differentially expressed genes were enriched in cell migration, angiogenesis, extracellular matrix organization, cell proliferation, T cell mediated cytotoxicity and collagen fibril organization (Fig. 4B). Furthermore, the Gene set enrichment analysis (GSEA) analysis demonstrated that the cell growth, cell migration, extracellular matrix organization, angiogenesis, collagen fibril organization and T cell mediated cytotoxicity were correlated with HNRNPC knockdown (Fig. 4C–H). Therefore, our data revealed that HNRNPC was involved in cell growth, cell migration, extracellular matrix organization and angiogenesis in NSCLC.

### HNRNPC knockdown attenuated the cell clonogenicity and proliferation in vitro

**Fig. 5:**
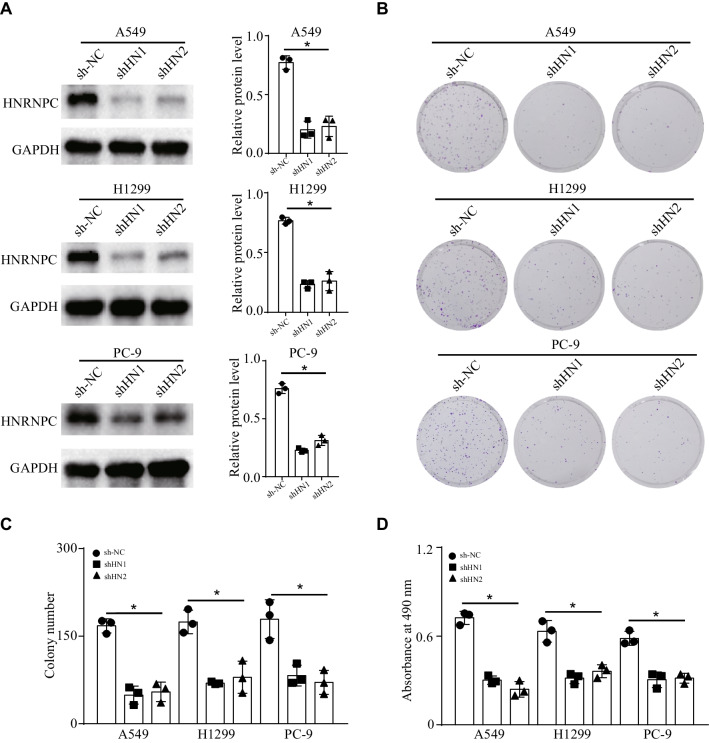
HNRNPC knockdown attenuated the cell clonogenicity and proliferation in vitro. **a** We established stable HNRNPC-knockdown cell lines (A549, H1299, and PC-9) by transfecting cells with the lentivirus. WB analysis confirmed that the expression of HNRNPC was inhibited after HNRNPC knockdown (*n* = 3). **b, c** clone formation assay revealed that HNRNPC knockdown attenuated the cell clonogenicity of NSCLC after 2 weeks (*n* = 3). **d** MTS assay showed that HNRNPC knockdown suppressed the proliferation of NSCLC cells after 24 h (*n* = 3). *P < 0.05

To validate the function of HNRNPC in NSCLC, we established stable HNRNPC-knockdown cell lines (A549, H1299, and PC-9) by transfecting cells with the lentivirus. WB analysis confirmed that the expression of HNRNPC was inhibited after HNRNPC knockdown (Fig. 5A). Furthermore, clone formation assay revealed that HNRNPC knockdown attenuated the cell clonogenicity of NSCLC after 2 weeks (Fig. 5B, C). In addition, MTS assay showed that HNRNPC knockdown suppressed the proliferation of NSCLC cells after 24 h (Fig. 5D). Thus, we confirmed that HNRNPC knockdown attenuated the cell clonogenicity and proliferation in vivo.

### HNRNPC knockdown impaired the cell invasion and migration in vitro

**Fig. 6:**
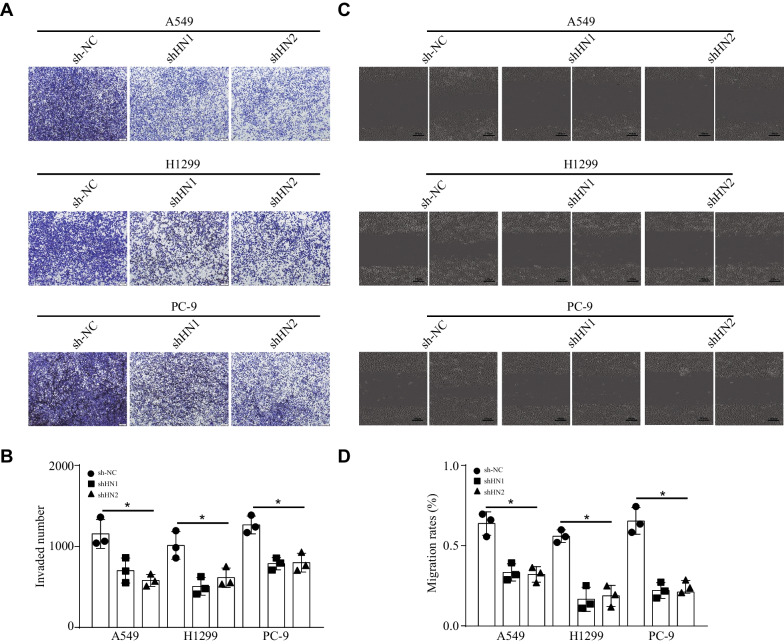
HNRNPC knockdown impaired the cell invasion and migration in vitro. **a, b** Transwell assay revealed that HNRNPC knockdown attenuated the cell invasion after 24 h (*n* = 3). **c, d** Scratch wound healing assay showed that HNRNPC knockdown inhibited the cell migration after 24 h (*n* = 3). *P < 0.05

To further evaluate the function of HNRNPC in NSCLC, transwell and scratch wound healing assays were used to accurately monitor cell invasion and migration. Transwell assay revealed that HNRNPC knockdown attenuated the cell invasion after 24 h (Fig. 6A, B). Similarly, scratch wound healing assay showed that HNRNPC knockdown inhibited the cell migration after 24 h (Fig. 6C, D). Overall, these findings demonstrated that HNRNPC knockdown impaired the cell invasion and migration in vitro.

### HNRNPC knockdown inhibited the tumor growth in vivo and was associated with CD8 + T cell infiltration in TIME

**Fig. 7:**
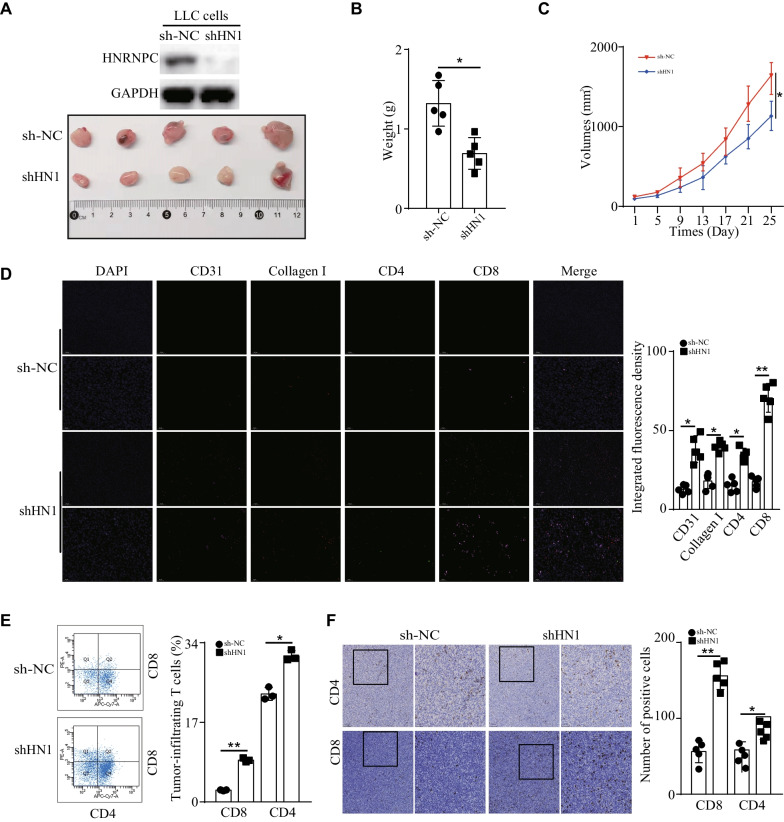
HNRNPC knockdown inhibited the tumor growth in vivo and was associated with CD8 + T cell infiltration in TIME. **a** In xenograft-bearing mouse models, mouse lung cancer cells (LLC) of stable HNRNPC-knockdown were subcutaneously injected into the left flank of nude mice. The expression of HNRNPC was inhibited after HNRNPC knockdown and the representative figure of tumors formed in each group was exhibited. **b, c** The tumor weight and tumor volumes were inhibited after HNRNPC knockdown. **d** In tumor xenografts, the fluorescence density of CD8 was significantly increased after HNRNPC knockdown. Moreover, the fluorescence densities of CD4, CD31 and collagen I were more than that of the control group. **e** The results of flow cytometry showed that the number of tumor-infiltrating CD8 positive T cells was significantly higher than that of the control group, along with elevated tumor-infiltrating CD4 positive T cells. **f** IHC staining of CD4 and CD8 was performed in tumor xenografts. Similarly, HNRNPC knockdown caused an obvious increase of tumor-infiltrating CD8 positive T cells, along with elevated CD4 positive T cells. *P < 0.05, **P < 0.01

The function of HNRNPC was subsequently investigated in vivo. In xenograft-bearing mouse models, mouse lung cancer cells (LLC) of stable HNRNPC-knockdown were subcutaneously injected into the left flank of nude mice. Tumor volumes were measured and recorded every 4 days. After 25 days, the tumor xenografts were harvested and processed. As was shown in Fig. 7A, the expression of HNRNPC was inhibited after HNRNPC knockdown and the representative figure of tumors formed in each group was exhibited (Fig. 7A). Furthermore, the tumor weight and tumor volumes were inhibited after HNRNPC knockdown, which revealed that HNRNPC knockdown inhibited the tumor growth in vivo (Fig. 7B, C). Next, to examine the relationship between HNRNPC and tumor microenvironment, IF was analyzed in tumor xenografts and the representative staining images of DAPI, CD4, CD8, CD31 and collagen I were shown in Fig. 7D. The results revealed that the fluorescence density of CD8 was significantly increased after HNRNPC knockdown. Moreover, the fluorescence densities of CD4, CD31 and collagen I were more than that of the control group (Fig. 7D). At the same time, the results of flow cytometry showed that the number of tumor-infiltrating CD8 positive T cells was significantly higher than that of the control group, along with elevated tumor-infiltrating CD4 positive T cells (Fig. 7E). In addition, IHC staining of CD4 and CD8 was performed in tumor xenografts. Similarly, HNRNPC knockdown caused an obvious increase of tumor-infiltrating CD8 positive T cells, along with elevated CD4 positive T cells (Fig. 7F). Therefore, our data revealed that HNRNPC knockdown inhibited the tumor growth in vivo and was associated with CD8 + T cell infiltration in TIME.

### HNRNPC knockdown suppressed the lung metastasis in vivo

**Fig. 8:**
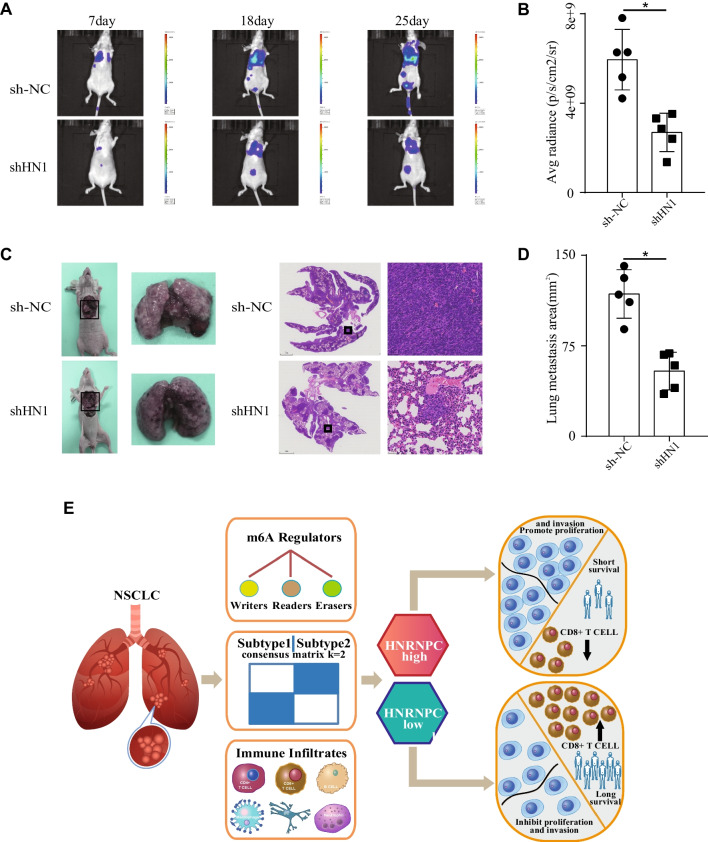
HNRNPC knockdown suppressed the lung metastasis in vivo. **a, b** Stable HNRNPC-knockdown cells (H1299) were transfected with luciferase plasmids and then injected into the tail veins of nude mice. The lung metastatic lesion was then assessed after 7, 18, 25 days. The results showed that HNRNPC knockdown effectively decreased the average radiance of lung metastatic lesion. **c, d** After 25 days, we used nude mouse lung metastases to make paraffin sections and perform HE staining. Similarly, HNRNPC knockdown caused a reduction of lung metastasis area. **e** We proposed a model for this study. First, the expression levels of m6A-related genes were correlated with prognosis and immunotherapy response of NSCLC. Next, the m6A regulator HNRNPC was selected as the most influential predictor for NSCLC, and HNRNPC predicted poor prognosis and correlated with tumor invasion and lymph node metastasis. Finally, HNRNPC knockdown inhibited the proliferation and invasion of NSCLC cells and was associated with CD8 + T cell infiltration in TIME. *P < 0.05

Next, to observe the role of HNRNPC in tumor metastasis in vivo, stable HNRNPC-knockdown cells (H1299) were transfected with luciferase plasmids and then injected into the tail veins of nude mice. The lung metastatic lesion was then assessed using bioluminescence imaging technology after 7, 18, 25 days. The results showed that HNRNPC knockdown effectively decreased the average radiance of lung metastatic lesion (Fig. 8A, B). After 25 days, we used nude mouse lung metastases to make paraffin sections and perform HE staining. Similarly, HNRNPC knockdown caused a reduction of lung metastasis area (Fig. 8C, D). In total, our results indicated that HNRNPC knockdown suppressed the lung metastasis in vivo.

Here, we proposed a model for this study (Fig. 8E). First, the expression levels of m6A-related genes were correlated with prognosis and immunotherapy response of NSCLC. Next, the m6A regulator HNRNPC was selected as the most influential predictor for NSCLC, and HNRNPC predicted poor prognosis and correlated with tumor invasion and lymph node metastasis. Finally, HNRNPC knockdown inhibited the proliferation and invasion of NSCLC cells and was associated with CD8 + T cell infiltration in TIME. In summary, this study indicates that m6A regulator HNRNPC, as a predictor of prognosis and immunotherapy response based on bioinformatics analysis, is related to proliferation and invasion of NSCLC cells.

## Discussion

Accumulating evidence has determined the significant role of m6A methylation in various cancers, which is mediated by m6A regulators, named writers, erasers and readers. As the m6A writer in NSCLC, METTL3 promotes drug resistance and metastasis of NSCLC by enhancing YAP activity [25]. The m6A demethylase ALKBH5 termed as eraser, inhibits NSCLC development and progression by reducing YAP activity [26]. The diverse functions of m6A regulators in NSCLC indicate that the mechanism of m6A methylation modification was complex. However, the prognostic power of the m6A regulators in NSCLC and the relationship between m6A regulators and TIME remains to be interpreted. It is unclear which m6A regulators are essential for NSCLC progression. The aim of this work was to excavate the role of m6A-related genes in the TIME and progression of NSCLC. In this paper, for the first time, the m6A-related genes with prognosis, PD-L1 and TIME in NSCLC were assessed. Furthermore, HNRNPC was considered as the most influential m6A regulator and associated with proliferation and invasion of NSCLC cells. We also confirmed that HNRNPC predicted poor prognosis and correlated with tumor invasion and lymph node metastasis in NSCLC patients. Targeting HNRNPC may provide promising therapeutic strategies for NSCLC.

Based on TCGA database, we analyzed the expression profiles of 22 m6A regulators in NSCLC and normal controls, and predicted the prognostic power of genes of interest. Our data showed that the expressions of 15 DEMGs were significantly different between NSCLCs and normal controls, including 4 down-regulated genes and 11 up-regulated ones. The heatmap illustrated the differential expression of 15 DEMGs could distinguish NSCLC samples from normal samples separately, indicating the significance of the 15 DEMGs in NSCLC. Consensus clustering is a class technique that facilitates possible clusters based on similar intrinsic features [16]. In our study, the NSCLC samples from TCGA database were regrouped by consensus clustering, which was in favor of personalized intervention and recommendations for NSCLC patients. Our data showed that based on comprehensive expression of the 15 DMEGs, the NSCLC samples were clustered into 2 subtypes. Survival analysis suggested that subtype 1 was correlated with preferentially longer survival time than subtype 2, revealing the expression of 15 DMEGs was associated with the prognosis of NSCLC patients.

Increasing evidence has demonstrated that TIME characterized by immune cell infiltration is closely related with prognosis of cancers [27, 28]. The fractions of infiltrated immune cells were predicted by CIBERSORT in each tumor sample based on the expression profiles of m6A-related genes. The distinct immune cell infiltration and immune score were obtained between 2 subtypes. The immune score was significantly higher in subtype1 group. It is reported that higher immune score was associated with longer survival time of tumor samples [29], which was consistent with our results that the survival time was significantly longer in subgroup1 by survival analysis. In addition, PD-L1 is an indicator for TIME and implicated in tumor immune escape [30]. The expression level of PD-L1 was significantly lower in tumor tissues compared with normal controls, while obviously higher in subtype 1 relative to subtype 2. PD-L1 expression was correlated with majority of the 15 m6A-related genes based on TCGA dataset, which was validated by GSE50081 dataset. All these suggested that m6A-related genes were closely associated with immune response and could play key roles in TIME of NSCLC.

In order to screen the prognostic biomarker for NSCLC, we established LASSO regression model for the 15 DMEGs in TCGA training dataset. LASSO algorithm allows for the most influential variable screening from all the variables simultaneously [31], which is more accurate than the traditional method. In this study, 5 of 15 genes (HNRNPA2B1, HNRNPC, IGF2BP1, METTL3 and RBM15B) were found to be the candidate prognostic biomarkers for NSCLC based on LASSO model. The predictive values of the five biomarkers were determined by ROC curve analysis. RS of the five genes was an independent factor for prognosis and was linked with subtypes and tumor recurrence.

In this study, METTL3 was found to be overexpressed in NSCLC samples based on TCGA dataset. The previous evidence has determined the upregulation of METTL3 in LUAD [32], which was consistent with our results. METTL3 has been found to play an oncogene role in liver cancer and leukemia, while it exerts tumor inhibitive effect on cervical cancer and breast cancer [32, 33]. METTL3 may play dual roles in various cancers. The study of Li et al. suggested that the reduced expression of METTL3 was accompanied with the activation of mTOR pathway, which was correlated with poor prognosis of renal cell cancer [34]. Besides, the expression of METTL3 was correlated with T cell proliferation and IL-7 sensitivity [35]. In lung cancer cells, METTL3 promotes the translation of EGFR and TAZ, the effector of Hippo pathway [32]. The depletion of METTL3 suppressed translation and oncogenesis of lung cancer [36]. Our data revealed that RS of the five genes was an independent risk factor for NSCLC prognosis. RS was negatively correlated with CD4 + T cells, while positively correlated with CD8 + T cells, suggesting that METTL3 might be a prognostic biomarker for NSCLC by regulating immune cell infiltration in TIME. The emerging evidence has proposed the oncofetal IGF2 mRNA binding protein 1 (IGF2BP1) as the cancer turnover regulator for its pivotal role in stabilizing the pro-oncogenic mRNA expression [37]. IGF2BP1 knockdown suppressed the proliferation and promoted apoptosis of NSCLC cells induced by high glucose [38]. Low IGF2BP1 was correlated with good outcome in LUAD patients [39]. In our study, the expression level of IGF2BP1 was obviously higher in NSCLC tissues compared with normal controls, which agreed with the previous studies. RNA binding motif protein 15 (RBM15) contributes to the progression of laryngeal squamous cell carcinoma [40]. Recent evidence has indicated that there is intimate relationship between RBM15 and immune signature in kidney renal cell carcinoma [41]. Our results showed that RS of the five m6A-related genes were correlated with immune cell infiltration in TIME. The immune related function of RBM15 was in agreement with the previous study.

Heterogeneous nuclear ribonucleoprotein A2B1 (HNRNPA2B1) and heterogeneous nuclear ribonucleoprotein C (HNRNPC) as the members of heterogeneous nuclear ribonucleoprotein family, have been found to be up-regulated in various types of cancers, such as liver cancer and glioblastoma [42–44]. In agreement with the previous reports, our data showed that the expressions of HNRNPC and HNRNPA2B1 were significantly higher in NSCLC samples than that in normal controls. It has been found that increased HNRNPA2B1 promotes the growth and mobility of ovarian cancer cells and matches along with poor prognosis of ovarian cancer patients [45]. Knockdown of HNRNPA2B1 pronouncedly reduced the migration and invasion of lung squamous cell carcinoma cell line DLKP-M [46]. HNRNPC was overexpressed in chemo-resistant gastric cancer (GC) cells and suggested as the prognostic biomarker for GC [47]. All these supported the potential of HNRNPA2B1 and HNRNPC as the prognostic indicators for NSCLC.

As discussed above, HNRNPC and RBM15B were correlated with PD-L1 both in training dataset and validation dataset. Due to the higher coefficient in RS formula, HNRNPC was considered as the most influential m6A regulator. HNRNPC is a conserved pre-mRNA-binding protein that is involved in multiple processes of gene expression, including transcription, mRNA splicing, translation, and genome stability. But in NSCLC, the specific functions of HNRNPC are still unknown. Moreover, it is unclear whether HNRNPC is essential for NSCLC progression, and its prognostic value are rarely reported. In our study, we found that the expression of HNRNPC was enhanced in human NSCLC tissues. More importantly, the patients with high levels of HNRNPC had poor prognosis and correlated with tumor invasion and lymph node metastasis, demonstrating that HNRNPC was a poor prognostic biomarker for NSCLC. The RNA-seq data showed that HNRNPC was involved in cell growth, cell migration, extracellular matrix organization, angiogenesis, collagen fibril organization and T cell mediated cytotoxicity in NSCLC. Therefore, we hypothesized HNRNPC in NSCLC acted as a potential tumor-promoting role by regulating cell proliferation, migration and tumor microenvironment. Proliferating Cell Nuclear Antigen (PCNA) and N-cadherin are known as the molecular markers used for cell proliferation and invasion measurement. In human NSCLC tissues, we found a positive expression correlation between HNRNPC and PCNA, along with a positive expression correlation between HNRNPC and N-cadherin, which revealed that HNRNPC was involved in cell proliferation and invasion. In vitro, the cell proliferation, clonogenicity, invasion and migration were suppressed after HNRNPC knockdown. In mouse xenograft models, the results were consistent with our in vitro data and showed that knockdown of HNRNPC inhibited the tumor growth and metastasis in vivo, suggesting an oncogene role of HNRNPC in NSCLC development.

CD8 is a transmembrane glycoprotein that is predominantly expressed on the surface of cytotoxic T cells, and can also be found on natural killer cells, cortical thymocytes, and dendritic cells. CD8 plays a role in T cell development and activation of mature T cells. CD4 is involved mainly in T helper cell development and activation, which is expressed on T helper cells, majority of thymocytes, monocytes, macrophages, and dendritic cells [48]. According to the RNA-seq analysis, we explored the relationship between HNRNPC and tumor-infiltrating CD8 or CD4 positive T cells. The results showed that knockdown of HNRNPC was significantly associated with high CD8 + T cell infiltration, along with lightly elevated CD4 + T cell infiltration. Thus, we confirmed that HNRNPC was related with CD8 + T cell infiltration in TIME of NSCLC. Meanwhile, as markers of vascular endothelial cells and fibroblasts, CD31 and collagen I were increased after HNRNPC knockdown, indicating that HNRNPC might also modulate angiogenesis and collagen fibril organization of tumor microenvironment. We hypothesized that HNRNPC promoted proliferation and invasion of NSCLC cells through regulating T cell infiltration, angiogenesis and collagen fibril organization. Notably, the specific action mechanism still needs future investigations. The further relationship between HNRNPC and tumor microenvironment in NSCLC will be conducted in the future research.

## Conclusions

In summary, this study indicates that m6A regulator HNRNPC, as a predictor of prognosis and immunotherapy response based on bioinformatics analysis, is related to proliferation and invasion of NSCLC cells.

## Supplementary Information

**Additional file 1.** The bioinformatics databases used in this study.

**Additional file 2.** The profile of the analyzed 994 NSCLC samples and their information.

**Additional file 3.** The expression value of 20 m6A-related genes in NSCLC samples and normal controls.

**Additional file 4.** The NSCLC samples were classified into subtype 1 and subtype 2.

**Additional file 5.** The proportions of total 22 types of immune cells were predicted.

**Additional file 6.** The immune scores of each tumor sample were calculated.

**Additional file 7.** Total 10 KEGG pathways were predicted to be related with high and low TIME.

**Additional file 8.** Correlation analysis between PD-L1 expression and DEMGs expression.

**Additional file 9.** The LASSO regression coefficient of five DEMGs.

**Additional file 10.** RS was calculated based on the LASSO regression coefficient and the expression level of optimal DEMGs.

**Additional file 11.** The fractions of six types of immune cells were predicted.

## Data Availability

All data generated and/or analyzed during this study are available from the corresponding author on reasonable request.

## Abbreviations

m6A: N6-methyladenosine
NSCLC: Non-small cell lung cancer
HNRNPC: Heterogeneous nuclear ribonucleoprotein C
TIME: Tumor immune microenvironment
GEO: Gene expression omnibus
GO: Gene ontology
KEGG: Kyoto encyclopedia of genes and genomes
WB: Western blot
IHC: Immunohistochemistry
IF: Immunofluorescence
PD-L1: Programmed cell death–ligand 1
LC: Lung cancer
LUAD: Lung adenocarcinoma
LUSC: Lung squamous cell carcinoma
m6A: N6-methyladenosine
RS: Risk score
FC: Flow cytometry
OD: Optical density
OS: Overall survival
GSCs: Glioblastoma stem cells
DEMGs: Differentially expressed methylation genes
ESTIMATE: Estimation of stromal and immune cells in malignant tumor tissues using expression data
TCGA: The Cancer Genome Atlas
LASSO: Least absolute shrinkage and selection operator
TIMER: Tumor immune estimation resource
ROC: Receiver operating characteristic
GSEA: Gene set enrichment analysis
RNA-seq: RNA sequencing
HPA: Human protein atlas
ANTs: Adjacent normal tissues
LLC: Lung cancer cells
IGF2BP1: IGF2 mRNA binding protein 1
RBM15: RNA binding motif protein 15
HNRNPA2B1: Heterogeneous nuclear ribonucleoprotein A2B1
GC: Gastric cancer
PCNA: Proliferating cell nuclear antigen
DAPI: 4,6-Diamidino-2-phenylindole
