# Supplementary material for: HNRNPC, a predictor of prognosis and immunotherapy response based on bioinformatics analysis, is related to proliferation and invasion of NSCLC cells
Source: Respir Res. 2022 Dec 19;23:362. doi: 10.1186/s12931-022-02227-y (PMC9761959; doi:10.1186/s12931-022-02227-y)
Supplement: Supplementary file 1 — Additional file 1. The bioinformatics databases used in this study. [file 12931_2022_2227_MOESM1_ESM.doc]

**Bioinformatics** databases used in this study.

| **Name** | **Keywords** | **Link** | **Precise Description** |
| --- | --- | --- | --- |
| TCGA | The Cancer Genome Atlas | https://portal.gdc.cancer.gov/ | The gene expression datasets of 585 LUAD samples and 550 LUSC samples were downloaded from TCGA. After checking the expression data and the clinical information, 994 NSCLC samples with complete clinical information were included in this study and set as the training dataset. |
| SVA package | SVA package version 3.38.0 in R3.6.1 | <http://www.bioconductor.org/packages/release/bioc/html/sva.html> | Since the LUAD data and LUSC data were derived from different batches, SVA package, which contains functions for removing batch effects and other unwanted variation, was utilized to process the 994 NSCLC samples. After SVA-based batch effects elimination, LUAD and LUSC samples were mixed together, indicating the batch effects have been removed. |
| GEO | Gene Expression Omnibus | <https://www.ncbi.nlm.nih.gov/geo/> | The GSE50081 dataset was downloaded from GEO database. This dataset included 181 NSCLC samples with clinical information. This dataset was sequenced based on the platform of GPL570 and served as the validation dataset. |
| Pheatmap | Pheatmap Version 1.0.8 in R3.6.1 | <https://cran.r-project.org/web/packages/pheatmap/index.html> | The expression profiles of 22 recognized m6A RNA methylation related genes were collected from TCGA dataset and their expression in NSCLC samples and normal counterparts were compared by t test in R 3.6.1. The differentially expressed methylation genes (DEMGs) were subjected to hierarchical clustering analysis according to centered pearson correlation algorithm, with the application of Pheatmap Version 1.0.8 in R3.6.1. The heatmap of the expression levels of DEMGs in the samples was shown in Figure 1B, and the distribution of expression levels in the tumor and control samples was shown in Figure 1C. |
| ConsensusClusterPlus package | ConsensusClusterPlus package version 1.54.0 in R 3.6.1 | <http://www.bioconductor.org/packages/release/bioc/html/ConsensusClusterPlus.html> | To further understand the biological features of DEMGs, the TCGA NSCLC samples were distinctly classified into two subtypes, defined as subtype 1 and subtype 2. The procedure was based on the expression level of the DEMGs using ConsensusClusterPlus package, which is an open-source software for unsupervised class discovery. |
| Survival package | Survival package version 2.41-1 in R 3.6.1 | <http://bioconductor.org/packages/survivalr/> | Kaplan-Meier survival analysis was performed to evaluate the correlation of survival time and different subtypes by using survival package. The clinical information of different subtypes was compared, such as age, gender, pathologic stage, metastasis, treatment, and recurrence. |
| CIBERSORT | CIBERSORT | <https://cibersortx.stanford.edu/> | The immune cell fractions of NSCLC samples were evaluated by CIBERSORT. Briefly, the RNA-seq data of TCGA NSCLC samples and a “signature matrix” containing signature genes for cell subsets of 22 types of immune cells were used as input. “Permutations” was set as 100 and the options of “disable quantile normalization” was checked. After running, the proportions of the immune cells for each sample were obtained. The proportions of different immune cells were compared between subtypes by t test in R 3.6.1 with P <0.05 as threshold. |
| ESTIMATE | Estimation of Stromal and Immune cells in Malignant Tumor tissues using Expression data | <https://sourceforge.net/projects/estimateproject/> | The ESTIMATE is a tool for predicting tumor purity using gene expression data, generating three scores, including stromal score, immune score, and ESTIMATE score. As described previously, the immune scores of each tumor sample were calculated by ESTIMATE package. The difference between immune scores was analyzed by t test in R 3.6.1 with P < 0.05 as the threshold. The subgroups were divided into high TIME group and low TIME group according to the distribution of immune scores of all samples. |
| GSEA | Gene Set Enrichment Analysis | <http://software.broadinstitute.org/gsea/index.jsp> | Based on the gene expression profiles of all the included tumor samples, the Kyoto Encyclopedia of Genes and Genomes (KEGG) pathways significantly related with TIME were analyzed by GSEA. The adjusted P value (FDR, false discovery rate) < 0.05 was set as the cutoff value. |
| LASSO | least absolute shrinkage and selection operator regression analysis by using lars package Version 1.2 in R 3.6.1 | <https://cran.r-project.org/web/packages/lars/index.html> | To screen the DEMGs with potential to predict prognosis, the DEMGs were subjected to LASSO in R 3.6.1. The optimal lambda value was determined by 10 cross-validations. Then, the optimal DEMGs related with prognosis was selected using multivariate Cox proportional hazard regression analysis and a prognostic signature of risk score (RS) was established as follows: RS = ∑Coefgenes ×Expgenes. Coefgenes indicates LASSO coefficient of target genes, Expgenes represents expression level of a given gene in TCGA dataset. |
| TIMER | Tumor Immune Estimation Resource | https://cistrome.shinyapps.io/timer/ | The effect of m6A related genes in immune cell infiltration was measured by online tool of TIMER, which contained 6 types of immune infiltrated cells, such as B cells, CD4+ T cells, CD8+ T cell, neutrophils, macrophage and dendritic cells. Then, the relationship between different types of infiltrated immune cells and RS value was calculated based on TCGA samples. |
